# The comings and goings of the design of the healthy eating policy in Peru: a comparative analysis of its regulatory documents

**DOI:** 10.17843/rpmesp.2022.394.11896

**Published:** 2022-12-23

**Authors:** Juan Alvarez-Cano, Victoria Cavero, Francisco Diez-Canseco

**Affiliations:** 1 CRONICAS, Centro de Excelencia en Enfermedades Crónicas, Universidad Peruana Cayetano Heredia, Lima, Perú. Universidad Peruana Cayetano Heredia CRONICAS Centro de Excelencia en Enfermedades Crónicas Universidad Peruana Cayetano Heredia Lima Perú

**Keywords:** Food Legislation, Food and Beverages, Food Advertising, Control of Product Advertising, Food Labelling

## Abstract

The sale of ultra-processed products has increased in Latin America in recent years, as well as the prevalence of overweight and obesity. In Peru, Law No. 30021 passed in an attempt to reduce overweight and obesity in children and adolescents; however, the development of this law was characterized by constant modifications to the documents prepared in this regard. This article aims to identify essential modifications in the documents elaborated by the Government and the Congress within the timeframe of Law No. 30021, particularly those regarding the regulation of food and non-alcoholic beverage advertising, advertising warnings and technical parameters of critical nutrients. The lack of timely scientific evidence, the opposition by the food industry and the lack of political consensus were the main reasons for the detected modifications, which shows the dynamism during the development of this policy.

## INTRODUCTION

According to the Pan American Health Organization (PAHO), the sale of ultra-processed products in Latin America increased 8.3% between 2009 and 2014, and an increase of 7.8% was estimated between 2015 and 2019 ^1^. Increased consumption of ultra-processed foods is associated with more overweight and obesity in all age groups ^2^ ^-^ ^4^.

Policies aimed at reducing the consumption of ultra-processed foods have been developed, such as increasing their taxes, regulating their advertising, using front labels on their packaging, and establishing technical parameters for critical nutrients such as sugar, sodium, and fat ^1^. In Latin America, countries such as Ecuador, Chile, Uruguay, Mexico, Brazil, and Peru have implemented one or more of these policies ^5^.

In Peru, seeking to reduce overweight and obesity in children and adolescents (CA) and prevent chronic non-communicable diseases, Law No. 30021 ^6^, “Law for the promotion of healthy eating for children and adolescents”, was approved in 2013. This law established, among other things, the regulation of food and non-alcoholic beverage (FNAB) advertising and the use of nutritional warnings (NW), for which it was necessary to define technical parameters for the maximum levels of sugar, sodium, saturated fats, and trans fats that a food or beverage could contain. Then, the Regulation of the Law ^7^ and the Nutritional Warnings Manual (NWM) were approved in 2017 and 2018 respectively, which established that NW would be black octagons placed on the front of the labels of products that exceeded the established parameters in order to inform the public about the high content of these nutrients ^8^. This marked the culmination of a policy development process that took several years and was characterized by constant changes and continuous opposition from the food industry and its political representatives.

Research on the development of public health policies is scarce in Peru. Regarding this particular policy, studies have focused on comparing different NW designs (e.g., Octagon vs. nutritional traffic light and GDA) ^9^ ^-^ ^10^ and the use of NW in consumption decisions ^11^ ^-^ ^12^; however, they have not addressed the design of this strategy or how it was modified over time. Analyzing the modifications made over the years in the documents that support public policies for regulating FNAB advertising and the use of NWs in Law No. 30021 would allow identifying the contents that remained and those that were changed, as well as to highlight the negotiations and struggles among the actors, and the complex dynamics behind their approval, in order to suggest improvements that would strengthen the current regulation. 

This article seeks to identify essential changes in the documents prepared by the Government and Congress within the framework of Law No. 30021, specifically in the areas of regulation of FNAB advertising, NW, and technical parameters of critical nutrients.

## METHODOLOGICAL APPROACH

From May 1 to June 15, 2022, the approved documents concerning Law No. 30021 ^6^ were collected from the web page of the Congress of the Republic of Peru (https://www.congreso.gob.pe/) and the Government of Peru (https://www.gob.pe/). The three main documents (Law No. 30021 ^6^, Regulation of the Law ^7^ and the NWM ^8^) were identified, as well as their bills and amending documents.

For the analysis of the documents, we considered the following types of legislation: *Bill*, which is a proposal submitted to a congressional commission. The commission debates the proposal and prepares a dictum with comments and suggestions. A substitute text is a document prepared on the basis of comments received in the plenary of Congress as a result of the discussion of a dictum. A *draft regulation* is a proposal made by a ministry that needs the approval of the Presidency of the Council of Ministers (PCM) to be published and reviewed by the population before being approved. Finally, a *modifying document* is a supreme decree approved that modifies a legal document, in this case, the Regulation of the Law and the NWM.

Each of the documents was read in its entirety by one of the researchers. Subsequently, each document was reviewed in detail to identify all the modifications. The most important thematic categories were identified based on the reading of these documents, and the corresponding texts of each document were added verbatim to an Excel file. Afterwards, the research team agreed on the most relevant categories related to the three prioritized topics (regulation of FNAB advertising, AW, and technical parameters of critical nutrients), so a second review of the contents of each document was carried out to identify the contents that were modified, as well as those that were maintained, according to the prioritized topics. Finally, the team agreed on the most relevant modifications in each of these topics.

We included the journals of the congressional session debates, news, and interviews to strengthen the analysis of the documents and to be able to understand the possible reasons for the modifications we identified. We included selected quotes from interviews with key actors (politicians, officials, researchers, civil society representatives and media) related to the design of the Law, which are part of a broader collection, in which a total of 25 key actors related to the design, implementation and/or oversight of the NW and Law No. 30021 ^13^ were interviewed. All interviews were conducted virtually, between January and September 2021. The qualitative data collection was approved by the Ethics Committees of the Universidad Peruana Cayetano Heredia and the University of North Carolina at Chapel Hill, USA. All participants gave informed consent for the interviews and their recording. The interviews were coded with the ATLAS.ti program, then the content was analyzed ^14^.

## RESULTS

### Description of the documents

Eighteen documents, published between 2012 and 2022, were identified, including approved documents, bills, congressional debate journals, substitute texts, congressional committee dictums and documents modifying the Law and NWM Regulations (Table 1). Of the three bills identified, we only included Bill 1038/2011-CR in the analysis because it was the only one that included the prioritized issues.

**Table 1:** Timeline of documents included in the review, indicating the contents of the topics prioritized during the analysis.

Year	Documents	Advertising Regulation	Technical Parameters	Advertising Warnings
2012	Bill 1038/2011-CR: Law on health promotion for the protection of consumers, children and adolescents. [Link]	X	X	X
	Dictum of the Committee on Consumer Protection and Regulatory Agencies of Public Utilities (Dictum). [Link]	X	X	X
2013	Journal of the debate of the plenary session of the congress. - 02/05/2013. [Link]	X	X	
	Substitute text for the dictum of the Committee on Consumer Protection and Regulatory Agencies of Public Utilities (Substitute text). [Link]	X	X	X
	Journal of the debate of the plenary session of the congress. - 09/05/2013. [Link]	X	X	
	Law 30021: Law for the promotion of healthy nutrition for children and adolescents.. [Link]	X	X	X
2014	1st draft of the Regulations of the Law for the Promotion of Healthy Eating - RM N° 321-2014-MINSA.. [Link]		X	
2015	Regulation of technical parameters - DS N° 007-2015-SA (Repealed). [Link]		X	
2016	2nd draft of the Regulations of the Law on the Promotion of Healthy Eating - RM N°524-2016-MINSA.. [Link]	X	X	X
2017	Regulation of the Law on Promotion of Healthy Eating - DS N° 017-2017-SA. [Link]	X	X	X
	Draft of the Advertising Warnings Manual - RM N° 683-2017-MINSA. [Link]			X
2018	Manual de Advertencias Publicitarias - DS N° 012-2018-SA. [Link]			X
2019	Supreme Decree modifying the Regulation and Manual of Advertising Warnings - DS N° 015-2019-SA. [Link]			X
2020	Supreme Decree extending the term set forth in sub-number 8.3 of the Advertising Warnings Manual. - DS N° 021-2020-SA. [Link]			X
2021	Supreme Decree extending for imported products the term set forth in sub-number 8.3 of the Advertising Warnings Manual. - DS N° 018-2021-SA. [Link]			X
2022	Supreme Decree extending the term that allows the use of stickers with advertising warnings for imported products and for micro and small companies as provided for in sub-numerals 8.3 and 8.5 of the Advertising Warnings Manual. - DS N° 005-2022-SA. [Link]			X

Bills not included: 774/2011-CR (Link) and 775/2011-CR (Link)

### Regulation of advertising of food and non-alcoholic beverages

Several articles underwent no or minimal modifications. 

Some of the advertising regulations that were maintained included the prohibition of showing rations that are not appropriate for the situation or age of the target public: “generating expectations referring to the fact that its ingestion provides a sensation of superiority or that its lack of ingestion is perceived as a situation of inferiority”, “representing social stereotypes or originate prejudices or any type of discrimination, linked to its ingestion”, among others.

**Figure 1:**
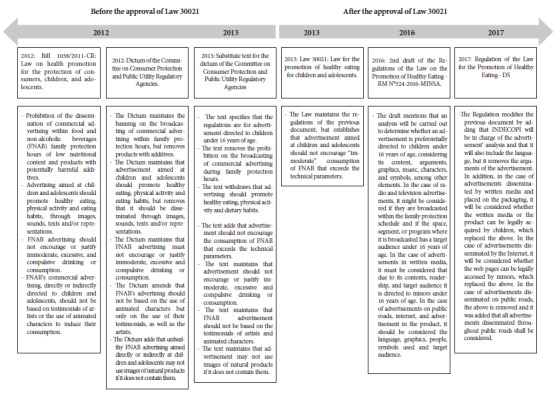
Changes in advertising regulations.

On the other hand, Figure 1 describes the articles that were modified, withdrawn, or added during the development of the documents. One of the most important modifications, prior to the approval of the bill, was the removal of the prohibition on high critical nutrient FNABs being advertised during family protection time. Some interviewees commented that this potential ban generated strong opposition from the industry and the media because of the potential economic losses it would cause and eventually led to discussions with congressmen from various parties to block the proposal. Interviewees and congressional debate journals show that congressmen from different parties opposed this article, calling for the elimination of this ban, which was eventually withdrawn.

*When it passed to the plenary [of Congress], they [the industry] took it seriously and said “no, this is dangerous”. And then the industry began to move, to move its influence. For several weeks industry representatives were in Congress, talking with one and another, trying to block the initiative (Congressman 2011-2016).*

Another important modification, prior to the approval of the Law, was that the regulation prohibiting the use of animated or fictitious characters in advertising was relaxed, prohibiting only their testimonials (Figure 1). 

No significant changes were made to the advertising regulation in the Regulation itself, its bills or in the NWM.

### Technical Parameters

Table 2 details the technical parameters found in the documents included in this section.

**Table 2:** Changes in technical parameters.

Documents		Article or provision	Phases	Type of food	Total sugar	Saturated fats	Sodium
Bill 1038 / 2011 - CR Law on health promotion for the protection of consumers, children and adolescents*.		Article 2: Definitions	One phase	Solid	Greater than or equal to 5g /100g	Greater than or equal to 1.5g / 100g	Greater than or equal to 300mg / 100g
				Liquid	Greater than or equal to 2.5g /100ml	Greater than or equal to 0.75g / 100ml	Greater than or equal to 300mg / 100ml
Dictum of the Committee on Consumer Protection and Regulatory Agencies of Public Utilities*.		Article 3: Glossary	One phase	Solid	Greater than or equal to 5g / 100g	Greater than or equal to 1.5g / 100g	Greater than or equal to 300mg / 100g
				Liquid	Greater than or equal to 2.5g /100ml	Greater than or equal to 0.75g / 100ml	Greater than or equal to 300mg /100ml
Substitute text for the dictum of the Committee on Consumer Protection and Public Utility Regulatory Agencies		1st transitory complementary provision: On the regulation of technical parameters	Parameters are withdrawn (The document indicates that the technical parameters will be described in the regulations).				
Law Nº 30021: Law for the promotion of healthy nutrition for children and adolescents.		1st transitory complementary provision: On the regulation of technical parameters	Parameters are withdrawn (The document indicates that the technical parameters will be described in the regulations).				
1st draft of the Regulations of the Law on the Promotion of Healthy Eating-RM N°321-2014-MINSA**.		Article 4: Of the technical parameters.	One phase	Non-alcoholic beverages	Greater than or equal to 8.1g /100ml	Greater than or equal to 5.3g / 100ml	Greater than or equal to 540mg / 100ml
				Solid food in general	Greater than or equal to 12.5g /100g	Greater than or equal to 5.3g / 100g	Greater than or equal to 540mg / 100g
				Cereals and derivatives	Greater than or equal to 12.5g / 100g	Greater than or equal to 3.4g / 100g	Greater than or equal to 540mg / 100g
				Cakes, biscuits, and cookies	Greater than or equal to 19.6g / 100g	Greater than or equal to 9.8g / 100g	Greater than or equal to 540mg / 100g
				Snacks	Greater than or equal to 12.5g / 100g	Greater than or equal to 7g / 100g	Greater than or equal to 540mg / 100g
Regulation of the technical parameters - DS N° 007-2015-SA (Repealed)		Article 4: Technical parameters on processed foods and non-alcoholic beverages regarding sugar, salt, and saturated fat content.	One phase	Solid	Equal to or less than 5 g / 100g	Equal to or less than 1.5g / 100g	Equal to or less than 300 mg / 100g
				Beverage	Equal to or less than 2.5g / 100ml	Equal to or less than 0.75g/100ml	Equal to or less than 300mg / 100ml
2nd draft of the Regulation of the Law on the Promotion of Healthy Eating-RMN° 524-2016-MINSA***.		Annex: Technical parameters on food and non-alcoholic beverages with high sugar, sodium and saturated fat content referred to in Law No. 30021.	One phase	Solid	Greater than or equal to 10% of the total Kcal	Greater than or equal to 10% of the total Kcal	Greater than or equal to 1 mg / Kcal
				Liquid	Greater than or equal to 10% of the total Kcal	Greater than or equal to 10% of the total Kcal	Greater than or equal to 1 mg / Kcal
Regulation of the Law on Promotion of Healthy Eating-DSN° 017-2017-SA****.		Article 4: Technical parameters on processed foods regarding the content of sugar, sodium, saturated fat, trans fats.	Phase 1	Solid	Greater than or equal to 22.5g / 100g	Greater than or equal to 6g / 100g	Greater than or equal to 800mg / 100g
				Liquid	Greater than or equal to 6g / 100ml	Greater than or equal to 3g /100ml	Greater than or equal to 100mg /100ml
			Phase 2	Solid	Greater than or equal to 10g / 100g	Greater than or equal to 4g / 100g	Greater than or equal to 400mg /100g
				Liquid	Greater than or equal to 5g /100ml	Greater than or equal to 3g / 100ml	Greater than or equal to 100mg / 100ml

* Parameters based on the “Recommendation of the PAHO Expert Consultation on the Promotion and Advertising of Foods and Non-alcoholic Beverages to Children in the Region of the Americas”. [Link].

** Parameters based on the study of the National Food and Nutrition Center (CENAN): “Percentile values of sugar, fat and sodium content in industrialized foods according to labeling sold in Lima”. [Link].

*** Parameters based on the “PAHO Nutrient Profile Model.” [Link].

**** Parameters based on Chilean regulations [Link].

One of the most important modifications, which occurred before its approval, was the removal of the technical parameters from the text of the Law, indicating that they should be defined later, in its Regulations. One interviewee commented that representatives of the Ministry of Health (MINSA) suggested that the technical parameters be removed from the Law in order to work on them in greater detail in the Regulations, and that a PAHO representative suggested specifying that the parameters be elaborated based on PAHO/WHO recommendations, to avoid potential “arbitrariness” on the part of MINSA. Likewise, some interviewees and congressional debate journals pointed out that the technical parameters initially proposed in the Law “did not have scientific evidence” and were “only” recommendations of a working group of experts. In addition, congressmen from different parties suggested including the technical parameters in the Regulation.

*We had the votes to win this [pass the Law] in Congress, but I also needed the support from the Executive (...) but the Executive began to call me to tell me to leave the technical parameters for the Regulation (...) that this has to be analyzed calmly, with the health authorities (...) and that these parameters are (only) from a document of experts (Congressman 2011-2016).*

In addition, after the approval of the Law, the technical parameters changed during the elaboration of different documents by MINSA, some of which were submitted for public consultation (RM N° 321-2014-MINSA, DS N° 007-2015-SA, RM N°524-2016-MINSA and DS N° 017-2017-SA). The interviewees commented that these changes were due to the lack of official technical parameters from PAHO, the lack of political consensus and the lack of economic resources to conduct research that would allow the development of their own technical parameters.

In 2015, WHO published the Nutrient Profile Model, which, according to a former MINSA official, was included in the 2017 Regulation proposal. However, the official indicated that this proposal did not achieve the approval of the PCM, so it was decided to opt for the Chilean technical parameters that were more lax.

*The PCM was never going to approve a proposal for a Supreme Decree [Regulation] with PAHO parameters. So, that is why this gradual process [Chilean parameters] was put in place, that is, either we move forward or we move forward (MINSA official).*

### Advertising Warnings

**Figure 2:**
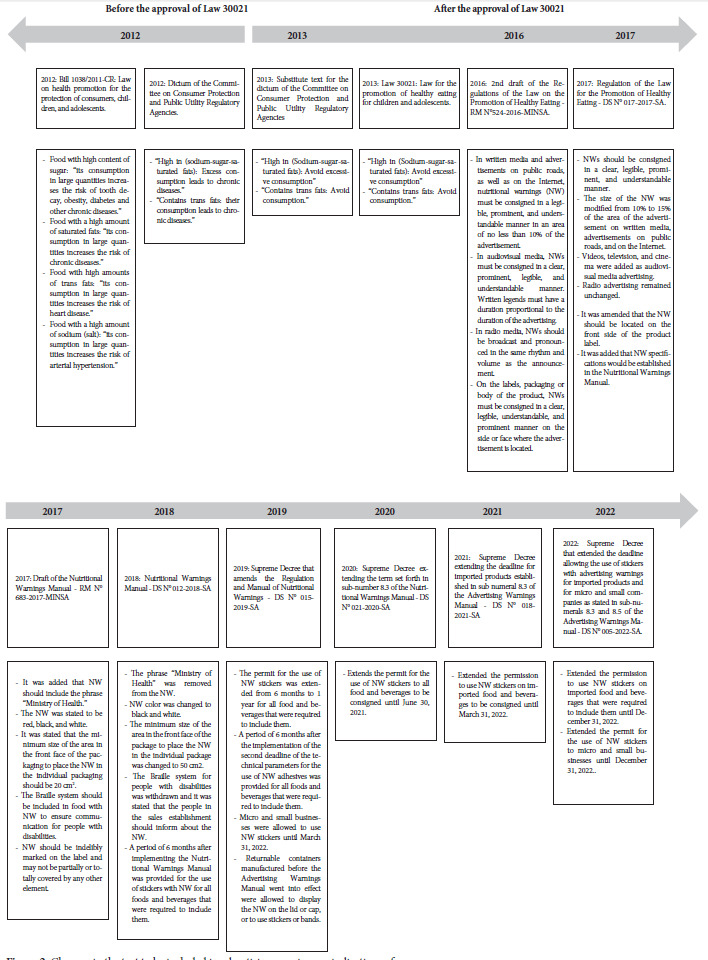
Changes in the text to be included in advertising warnings or indications of use

Figure 2 describes the changes in NWs found in the reviewed documents.

One of the most important modifications was found in the NW text. In Bill 1038/2011-CR the text was extensive, which indicated the consequences of consuming high amounts of critical nutrients, while the approved Law had significantly less text.

Subsequently, several modifications were made between the NWM bill and the approved NWM, such as changing the octagon from red to black, removing the phrase “Ministry of Health” from the NW and changing the minimum size that the package had to have for placing the NW, from 20 cm^2^ to 50 cm^2^ on the front face of the package. A MINSA official pointed out that the changes were made in response to the recommendations that MINSA received after the pre-publication of the NWM project, as well as due to negotiations in the PCM, where “not only technical aspects prevailed.” It was also pointed out that the change in the color of the octagon was due to the fact that some countries rejected the red color because it represented “danger” and constituted an “obstacle to trade.”

*The Warnings Manual has had technical aspects basically, but there have also been negotiations... No, they did not call it negotiations, but finally decisions of Senior Management and with a more political tinge, let’s say, right? In other words, not everything was finally resolved by the technical team (MINSA official).*

In addition, although neither the Law nor its Regulations mentioned it, the NWM stated a six-month deadline to place the NWs with stickers. Moreover, this time was extended in the subsequent four approved amending documents between 2019 and 2022. The use of stickers and the extension was due to requests from the industry, which pointed out that: placing the octagons on the labels was costly, octagons could not be required on imported products, other countries with NW allowed their use indefinitely, and that this was due to the pandemic.

*I was in a meeting with the industry, and they told us that it was very expensive to put the [octagon] label on the packaging (Civil society representative).*

*Shortly [after] the pandemic started, the industry took advantage and asked the Ministry of Health to give them one more year to use stickers [stickers] instead of having the seal printed on the packaging, right? And it was accepted (Independent researcher).*

Finally, continuous modifications and expansions were identified in the list of products exempted from using NWs, since they were first mentioned in the substitute text, prior to the approval of the Law. The first exception to NWs was for FNABs in their natural state, not subjected to industrialization processes. Subsequently, more exceptions were added in the documents prepared between 2014 and 2022: foods for primary or minimal processing, foods for culinary preparation, culinary ingredients, breast milk substitutes, foods for special diets subject to the *Codex Alimentarius* and cereal-based baby foods for children over 2 years of age that do not contain added sugars.

## DISCUSSION

This article shows the most important changes, during a decade, in the regulation of FNAB advertising, NW, and the technical parameters of critical nutrients in Peru. The identified changes reveal the dynamic nature of the development of health policies ^15^, but also that a constant in the development of this policy was the opposition of the industry, which influenced the Congress and the Executive to delay its implementation and make the regulation more flexible.

The intervention of the food industry in the development of public policies is frequent in Latin America, where their economic power allows them to influence decision-makers to delay, modify or deny regulations ^16^. Evidence suggests that they use similar strategies in different countries of the region as in the case of Chile, Ecuador, and Mexico where the industry negotiated with congressmen and government decision-makers ^16^ ^,^ ^17^.

During the design process of the Law, the regulation of ABNA advertising was the issue that suffered the greatest opposition from the industry and the media, causing several modifications. The removal of the ban on the use of cartoons or fictitious cartoons in ABNA advertising was an important loss, because it is a common strategy to attract children in unhealthy products ^18^. In the opinion of some, these modifications weakened the regulation, but allowed sufficient support in Congress to pass the Law. Nevertheless, countries such as Chile and Mexico were able to maintain prohibitions on the use of cartoons or animated characters on the packaging of products with one or more octagons ^17^ ^,^ ^19^ ^,^ ^20^, a regulation that could also be implemented in Peru.

We identified that a major problem for the definition and subsequent use of technical parameters in NWs was the Law’s provision to require WHO/PAHO parameters at a time when they were not available. Although the authors of the Law did this to prevent the use of parameters favorable to industry rather than to the health of the population, the lack of official WHO/PAHO parameters at the time the Law passed was used as an argument by the opposition to prevent its application. Likewise, we identified that the lack of consensus within the PCM ended with the approval of technical parameters considered by the advocates of the Law as “more flexible” than those proposed by PAHO/WHO (nutrient profile) ^21^ ^,^ ^22^. This situation shows the limitations to ensure the implementation of public policies when the necessary technical support is not yet available. Moreover, the approval of the Regulation with parameters different from those of WHO/PAHO shows that, even with the information required in the legislation (in this case, the WHO/PAHO parameters indicated in the Law), the approval of policies requires consensus among the different political actors.

The advertising warnings were another point of controversy in the development of the policy, expressed both in the lack of consensus and in constant changes in design. One of the most important changes was the modification of the minimum size that the label had to have for the placement of NWs. This allowed many products with an area of less than 50 cm^2^ not to carry octagons ^23^ and others to slightly reduce the size of their packaging to avoid carrying them, thus preventing the usual consumers of these products, i.e. children, from making more informed purchasing decisions. In addition, the Mexican experience has shown that it is possible to indicate the number of octagons in a product, no matter how small its packaging may be.

Regarding design changes, initially, several promoters of the use of warnings supported the use of the nutritional traffic light ^24^ ^,^ ^25^; however, after the appearance of the octagon, they changed their position in favor of the latter ^26^. This reflects the fact that scientific evidence may vary over time, which means that policies can be improved and should be evaluated ^27^.

The changes described above are evidence of the development of the three prioritized components, whose changes are explained not only by technical reasons but also by political and economic negotiations. These changes meant that the implementation of these components was delayed for up to six years, as was the case with the application of the NWs. In addition, we found that the finally approved documents (Law No. 30021, Regulations and NWM) ended up being more flexible and potentially less effective. Such is the case of the advertising regulations, where elements that the authors of the Law considered fundamental were removed; or in the case of NWs, where features that, such as the phrase “Ministry of Health,” could have given greater strength to the warnings, but were eliminated. However, while some of the modifications weakened the policy and reduced its impact, these also made it possible for it to ultimately be approved and implemented.

Likewise, we found that a common and transversal limitation of the analyzed components was the lack of local research to support the decisions of the actors in favor of the Law. For example, greater scientific production in the country would have helped to provide better support for the importance of its implementation and use it as an argument against opponents who argued that the law was unnecessary. Moreover, ongoing research and evaluations of these policies, both in Peru and in the region ^28^ ^,^ ^29^, should be the input for making improvements and strengthening them, bringing them closer to achieving their objectives.

The limitations of the study include the fact that we could only analyze publicly available documents, since other versions with possible modifications of interest may not have been accessed; we also analyzed the documents exclusively from a public health perspective, and did not interpret the texts at the legal level.

In conclusion, in light of its regulatory documents, the design process of Peru’s current healthy eating policy was affected by constant delays and significant regulatory changes, which could have delayed and reduced its impact, and which would be associated with the opposition and influence of the food industry, the lack of consensus among political actors, and the lack of timely scientific evidence.
