# Supplementary material for: The comings and goings of the design of the healthy eating policy in Peru: a comparative analysis of its regulatory documents
Source: Rev Peru Med Exp Salud Publica. 2022 Dec 23;39(4):480–8. doi: 10.17843/rpmesp.2022.394.11896 (PMC11397771; doi:10.17843/rpmesp.2022.394.11896)
Supplement: Translation

# Translation: Idas y venidas del diseño de la política de alimentación saludable en el Perú: análisis comparativo de sus documentos regulatorios

**Authors:** Juan Alvarez-Cano, Victoria Cavero, Francisco Diez-Canseco

**Affiliations:** 1 CRONICAS, Centro de Excelencia en Enfermedades Crónicas, Universidad Peruana Cayetano Heredia, Lima, Perú.

**Keywords:** Legislación Alimentaria, Alimentos y Bebidas, Publicidad de Alimentos, Control de la Publicidad de Productos, Etiquetado de Alimentos

## Abstract

La venta de productos ultraprocesados y el sobrepeso y la obesidad han aumentado en América Latina en los últimos años. En el Perú, se aprobó la Ley N° 30021 en busca de reducir el sobrepeso y la obesidad en niños y adolescentes, sin embargo, el desarrollo de esta Ley se caracterizó por tener continuas modificaciones en los diversos documentos elaborados. El propósito de este artículo es identificar cambios esenciales en los documentos elaborados por el Gobierno y el Congreso en el marco de la Ley N° 30021, específicamente en los temas de regulación de la publicidad de alimentos y bebidas no alcohólicas, advertencias publicitarias y parámetros técnicos de nutrientes críticos. Los cambios identificados en los diferentes documentos muestran el dinamismo en el desarrollo de esta política donde la falta de evidencia científica oportuna, la oposición de la industria alimentaria y la falta de consenso político fueron los principales motivos.

## INTRODUCCIÓN

Según la Organización Panamericana de la Salud (OPS), la venta de productos ultraprocesados en América Latina aumentó 8,3% entre 2009 y 2014, y se estimaba un aumento del 7,8% entre 2015 y 2019 ^1^. El mayor consumo de alimentos ultraprocesados está asociado a más sobrepeso y obesidad en todos los grupos etarios ^2^ ^-^ ^4^.

Para enfrentar este problema de salud pública, se han desarrollado políticas dirigidas a reducir el consumo de alimentos ultraprocesados, como aumentar sus impuestos, regular su publicidad, utilizar etiquetas frontales en sus empaques, y establecer parámetros técnicos para nutrientes críticos como el azúcar, el sodio y las grasas ^1^. En América Latina, países como Ecuador, Chile, Uruguay, México, Brasil y Perú han implementado una o más de estas políticas ^5^. 

En Perú, buscando reducir el sobrepeso y la obesidad en niños, niñas y adolescentes (NNA) y prevenir las enfermedades crónicas no transmisibles, se aprobó la Ley N° 30021 ^6^, «Ley de promoción de la alimentación saludable para niños, niñas y adolescentes», en 2013. Esta ley establecía, entre otras cosas, la regulación de la publicidad de alimentos y bebidas no alcohólicas (ABNA) y el uso de advertencias publicitarias (AP), para las cuales fue necesario definir parámetros técnicos para los niveles máximos de azúcar, sodio, grasas saturadas y grasas trans que podría llevar un alimento o bebida. Luego, en 2017 y 2018 respectivamente, se aprobaron el Reglamento de la Ley ^7^ y el Manual de Advertencias Publicitarias (MAP), en el que se estableció que las AP serían octógonos negros colocados en la parte frontal de las etiquetas de los productos que excedían los parámetros establecidos para informar al público sobre el alto contenido de estos nutrientes ^8^. Así se culminó un proceso de desarrollo de la política que tomó varios años, y que estuvo caracterizado por cambios constantes y una continua oposición de la industria alimentaria y sus representantes políticos. 

La investigación del desarrollo de políticas públicas en salud es escasa en Perú. Respecto a esta política en particular, los estudios se han centrado en comparar distintos diseños de AP (ej. Octógono vs semáforo nutricional y GDA) ^9^ ^-^ ^10^ y el uso de las AP en las decisiones de consumo ^11^ ^-^ ^12^; sin embargo, no han abordado su diseño ni cómo se modificó en el tiempo. Analizar las modificaciones realizadas a lo largo de los años en los documentos que sustentan las políticas públicas de regulación de la publicidad de ABNA y el uso de las AP de la Ley N° 30021 permitiría identificar los contenidos que permanecieron y aquellos que fueron cambiados, así como evidenciar las negociaciones y pugnas entre los actores, y la compleja dinámica detrás de su aprobación, para así sugerir mejoras que fortalezcan la regulación vigente. 

Este artículo busca identificar cambios esenciales en los documentos elaborados por el Gobierno y el Congreso en el marco de la Ley N° 30021, específicamente en los temas de regulación de la publicidad de ABNA, AP y parámetros técnicos de nutrientes críticos.

## ABORDAJE METODOLÓGICO

Del 1 de mayo al 15 de junio del 2022, se recopilaron los documentos aprobados concernientes a la Ley N° 30021 ^6^ de la página web del Congreso de la República del Perú (https://www.congreso.gob.pe/) y del Gobierno del Perú (https://www.gob.pe/). Se identificaron los tres documentos principales (Ley N° 30021 ^6^, Reglamento de la Ley ^7^ y Manual de Advertencias Publicitarias (MAP) ^8^), así como sus proyectos y documentos modificatorios.

Para el análisis de los documentos se tuvo en consideración los siguientes tipos de legislaciones: *Proyecto de Ley*, que es una propuesta presentada en una comisión del Congreso. La comisión debate la propuesta y elabora un dictamen con comentarios y sugerencias. Un texto sustitutorio es un documento elaborado en base a comentarios recibidos en el pleno del Congreso a raíz de la discusión de un dictamen. *Un proyecto de reglamento* es una propuesta realizada por algún ministerio que necesita la aprobación de la Presidencia del Consejo de Ministros (PCM) para ser publicada y revisada por la población antes de aprobarse. Finalmente, un *documento modificatorio* es un decreto supremo aprobado que modifica un documento legal, en este caso, del Reglamento de la Ley y del MAP.

Cada uno de los documentos identificados fue leído completamente por uno de los investigadores. Posteriormente, se realizó una revisión detallada de cada documento para identificar todas las modificaciones a los textos. En base a la lectura de estos documentos, se identificaron las categorías temáticas más importantes y se fue agregando a una matriz en Excel, de manera literal, los textos correspondientes de cada documento. Después, se acordó con el equipo de investigación las categorías más relevantes relacionadas con los tres temas priorizados (regulación de la publicidad de ABNA, AP y parámetros técnicos de nutrientes críticos), por lo que se realizó una segunda revisión de los contenidos de cada documento para identificar los contenidos que se modificaron, así como los que se mantuvieron, según los temas priorizados. Finalmente, se acordaron en equipo cuáles eran las modificaciones más relevantes en cada uno de estos temas. 

Para enriquecer el análisis de los documentos y poder conocer las posibles razones de las modificaciones que identificamos se incluyeron los diarios de los debates de sesión del Congreso, noticias y entrevistas. Se incluyeron citas seleccionadas de entrevistas con actores clave (políticos, funcionarios, investigadores, representantes de sociedad civil y medios de comunicación) relacionados al diseño de la Ley, que son parte de una recolección más amplia, en la que se entrevistó a un total de 25 actores clave relacionados al diseño, implementación y/o fiscalización de las AP y Ley N° 30021 ^13^. Todas las entrevistas se realizaron de manera virtual, entre enero y setiembre del 2021. La recolección de datos cualitativos fue aprobada por los Comités de Ética de la Universidad Peruana Cayetano Heredia y de la Universidad de Carolina del Norte en Chapel Hill, en Estados Unidos. Todos los participantes dieron su consentimiento informado para las entrevistas y su grabación. Las entrevistas se codificaron con el programa ATLAS.ti y se realizó un análisis de contenido ^14^.

## RESULTADOS

### Descripción de los documentos

Se identificaron 18 documentos, publicados entre 2012 y 2022, entre documentos aprobados, proyectos, diarios de debates del Congreso, textos sustitutorios, dictámenes de comisiones del Congreso y documentos modificatorios del Reglamento de la Ley y el MAP (Tabla 1). De los tres proyectos de Ley identificados, el análisis solo incluyó el proyecto de Ley 1038/2011-CR por ser el único que incluía los temas priorizados.

**Tabla 1:** Línea del tiempo de documentos incluidos en la revisión, indicando sus contenidos de los temas priorizados en el análisis.

Año	Documentos	Regulación de la Publicidad	Parámetros Técnicos	Advertencias Publicitarias
2012	Proyecto de ley 1038/2011-CR: Ley de promoción de la salud para la protección de los consumidores niños, niñas y adolescentes. [Link]	X	X	X
	Dictamen de la Comisión de Defensa del Consumidor y Organismos Reguladores de los Servicios Públicos (Dictamen). [Link]	X	X	X
2013	Diario del debate de la sesión del pleno del congreso - 02/05/2013. [Link]	X	X	
	Texto sustitutorio del dictamen de la Comisión de Defensa del Consumidor y Organismos Reguladores de los Servicios Públicos (Texto sustitutorio). [Link]	X	X	X
	Diario del debate de la sesión del pleno del congreso - 09/05/2013. [Link]	X	X	
	Ley 30021: Ley de promoción de la alimentación saludable para niños, niñas y adolescentes. [Link]	X	X	X
2014	1er proyecto del Reglamento de la Ley de promoción de la alimentación saludable - RM N° 321-2014-MINSA. [Link]		X	
2015	Reglamento de los parámetros técnicos - DS N° 007-2015-SA (Derogado). [Link]		X	
2016	2do proyecto del Reglamento de la Ley de promoción de la alimentación saludable - RM N°524-2016-MINSA. [Link]	X	X	X
2017	Reglamento de la Ley de promoción de la alimentación saludable - DS N° 017-2017-SA. [Link]	X	X	X
	Proyecto del Manual de Advertencias Publicitarias - RM N° 683-2017-MINSA. [Link]			X
2018	Manual de Advertencias Publicitarias - DS N° 012-2018-SA. [Link]			X
2019	Decreto Supremo que modifica el Reglamento y Manual de Advertencias Publicitarias - DS N° 015-2019-SA. [Link]			X
2020	Decreto Supremo que amplía el plazo dispuesto en el subnumeral 8.3 del Manual de Advertencias Publicitarias - DS N° 021-2020-SA. [Link]			X
2021	Decreto Supremo que amplía para los productos importados el plazo dispuesto en el subnumeral 8.3 del Manual de Advertencias Publicitarias - DS N° 018-2021-SA. [Link]			X
2022	Decreto Supremo que amplía el plazo que permite el uso de adhesivos con las advertencias publicitarias para los productos importados y para las micro y pequeñas empresas dispuesto en los subnumerales 8.3 y 8.5 del Manual de Advertencias Publicitarias - DS N° 005-2022-SA. [Link]			X

Proyectos de ley no incluidos: 774/2011-CR (Link) y 775/2011-CR (Link)

### Regulación de la publicidad de alimentos y bebidas no alcohólicas

Varios artículos no sufrieron modificaciones o éstas fueron mínimas. Algunas de las regulaciones de la publicidad que se mantuvieron fueron la prohibición de mostrar porciones no apropiadas para la situación ni la edad del público objetivo; de «generar expectativas referidas a que su ingesta proporcione sensación de superioridad o que su falta de ingesta se perciba como una situación de inferioridad»; «representar estereotipos sociales o que originen prejuicios o cualquier tipo de discriminación, vinculados a su ingesta», entre otros.

Por otro lado, la Figura 1 describe los artículos que fueron modificados, retirados o agregados durante el desarrollo de los documentos. Una de las modificaciones más importantes, antes de la aprobación de la Ley, fue que se retiró la prohibición de que los ABNA altos en nutrientes críticos sean publicitados dentro del horario de protección familiar. Algunos entrevistados comentaron que esta posible prohibición generó una fuerte oposición de la industria y los medios de comunicación por las potenciales pérdidas económicas que provocaría, y dio lugar a conversaciones con congresistas de diversas bancadas para bloquear la propuesta. Los entrevistados y los diarios de debates del Congreso muestran que congresistas de distintos partidos se opusieron a este artículo, pidiendo que se eliminara esta prohibición, la cual fue finalmente retirada. 

*Al pasar al Pleno [del Congreso], ya lo tomaron en serio [la industria] y dijeron ‘no, esto es peligroso’. Y ahí la industria comenzó a moverse, a mover sus influencias. Durante varias semanas representantes de la industria estaban en el Congreso, conversando con uno y otro, tratando de bloquear la iniciativa* (Congresista 2011-2016).

**Figura 1:** Cambios en las regulaciones a la publicidad.

Otra modificación importante, previa a la aprobación de la Ley, fue que la prohibición de usar personajes animados o ficticios en la publicidad se flexibilizó, prohibiéndose únicamente sus testimonios (Figura 1). 

Tanto en el Reglamento y sus proyectos, como en el MAP, no se realizaron cambios significativos a la regulación de la publicidad. 

### Parámetros técnicos

La Tabla 2 detalla los parámetros técnicos presentes en los diferentes documentos incluidos en este apartado.

**Tabla 2:** Cambios en los parámetros técnicos.

Documentos	Artículo o disposición	Fases	Tipo de alimento	Azúcar total	Grasas saturadas	Sodio
Proyecto de ley 1038 / 2011 - CR Ley de promoción de la salud para la protección de los consumidores niños, niñas y adolescentes*.	Artículo 2: Definiciones	Una fase	Sólido	Mayor o igual a 5g /100g	Mayor o igual a 1.5g / 100g	Mayor o igual a 300mg / 100g
			Líquido	Mayor o igual a 2.5g /100ml	Mayor o igual a 0.75g / 100ml	Mayor o igual a 300mg / 100ml
Dictamen de la Comisión de Defensa del Consumidor y Organismos Reguladores de los Servicios Públicos*.	Artículo 3: Glosario	Una fase	Sólido	Mayor o igual a 5g / 100g	Mayor o igual a 1.5g / 100g	Mayor o igual a 300mg / 100g
			Líquido	Mayor o igual a 2.5g /100ml	Mayor o igual a 0.75g / 100ml	Mayor o igual a 300mg /100ml
Texto sustitutorio del dictamen dela Comisión de Defensa del Consumidor y Organismos Reguladores de los Servicios Públicos	1era disposición complementaria transitoria: Sobre la reglamentación de los parámetros técnicos	Se retiran los parámetros (El documento indica que los parámetros técnicos serán elaborados en el reglamento)				
Ley Nº 30021: Ley de promoción de la alimentación saludable para niños, niñas y adolescentes	1era disposición complementaria transitoria: Sobre la reglamentación de los parámetros técnicos	Se retiran los parámetros (El documento indica que los parámetros técnicos serán elaborados en el reglamento)				
1er proyecto del Reglamento de la Ley de promoción de la alimentación saludable-RM N°321-2014-MINSA**.	Artículo 4: De los parámetros técnicos.	Una fase	Bebidas no alcohólicas	Mayor o igula a 8.1g /100ml	Mayor o igual a 5.3g / 100ml	Mayor o igual a 540mg / 100ml
			Alimentos sólidos en general	Mayor o igual a 12.5g /100g	Mayor o igual a 5.3g / 100g	Mayor o igual a 540mg / 100g
			Cereales y derivados	Mayor o igual a 12.5g / 100g	Mayor o igual a 3.4g / 100g	Mayor o igual a 540mg / 100g
			Queques, bizcochos y galletas	Mayor o igual a 19.6g / 100g	Mayor o igual a 9.8g / 100g	Mayor o igual a 540mg / 100g
			Snacks	Mayor o igual a 12.5g / 100g	Mayor o igual a 7g / 100g	Mayor o igual a 540mg / 100g
Reglamento de los parámetros técnicos - DS N° 007-2015-SA (Derogado)	Artículo 4: De los parámetros técnicos sobre alimentos y bebidas no alcohólicas procesados referentes a los contenidos de azúcar, sal, grasas saturadas.	Una fase	Sólido	Igual o menor a 5 g / 100g	Igual o menor a 1.5g / 100g	Igual o menor a 300 mg / 100g
			Bebida	Igual o menor a 2.5g / 100ml	Igual o menor a 0.75g/100ml	Igual o menor a 300mg / 100ml
		2do proyecto del Reglamento de la Ley de promoción de la alimentación saludable-RMN° 524-2016-MINSA***.	Anexo: Parámetros técnicos sobre los alimentos y las bebidas no alcohólicas referente sal alto contenido de azúcar, sodio y grasas saturadas al que se refiere la Ley N° 30021.	Una fase	Sólido	Mayor o igual al 10% del total de Kcal	Mayor o igual al 10% del total de Kcal	Mayor o igual a 1 mg / Kcal
					Líquido	Mayor o igual al 10% del total de Kcal	Mayor o igual al 10% del total de Kcal	Mayor o igual a 1 mg / Kcal
Reglamento de la Ley de promoción de la alimentación saludable-DSN° 017-2017-SA****.	Artículo 4: De los parámetros técnicos sobre los alimentos procesados referentes al contenido de azúcar, sodio, grasa saturada, grasas trans.			1. a fase	Sólido	Mayor o igual a 22.5g / 100g	Mayor o igual a 6g / 100g	Mayor o igual a 800mg / 100g
					Líquido	Mayor o igual a 6g / 100ml	Mayor o igual a 3g /100ml	Mayor o igual a 100mg /100ml
		2 . a fase	Sólido	Mayor o igual a 10g / 100g	Mayor o igual a 4g / 100g	Mayor o igual a 400mg /100g
			Líquido	Mayor o igual a 5g /100ml	Mayor o igual a 3g / 100ml	Mayor o igual a 100mg / 100ml

*Parámetros basados en la "Recomendación de la consulta de expertos de la OPS sobre la promoción y publicidad de alimentos y bebidas no alcohólicas dirigida a los niños en la Región de las Américas”[Link].

** Parámetros basados en el estudio del Centro Nacional de Alimentación y Nutrición (CENAN): “Valores percentilares del contenido de azúcar, grasas y sodio en alimentos industrializados según etiquetado expendidos en Lima” [Link].

***Parámetros basados en el "Modelo de perfil de nutrientes de la OPS” [Link].

****Parámetros basados en la normativa chilena [Link].

Una de las modificaciones más importantes, ocurrida antes de su aprobación, fue el retiro de los parámetros técnicos del texto de la Ley, indicándose que deberían definirse posteriormente, en su Reglamento. Un entrevistado comentó que representantes del Ministerio de Salud (MINSA) sugirieron que los parámetros técnicos sean retirados de la Ley para trabajarlos con mayor detenimiento en el Reglamento, y que un representante de la OPS sugirió especificar que los parámetros sean elaborados en base a las recomendaciones de OPS/OMS, para evitar potenciales “arbitrariedades” del MINSA. Asimismo, algunos entrevistados y diarios de debates del Congreso señalaban que los parámetros técnicos inicialmente propuestos en la Ley “no tenían evidencia científica” y que eran “únicamente” recomendaciones de un grupo de trabajo de expertos. Además, congresistas de diversos partidos sugirieron incluir los parámetros técnicos en el Reglamento.

*Teníamos los votos para ganar esto [aprobar la Ley] en el Congreso, pero yo necesitaba el respaldo también del Ejecutivo (…) pero el Ejecutivo empezó a llamarme para decir que deje los parámetros técnicos para el Reglamento (…) que eso hay que analizarlo con calma, con las autoridades de salud (…) y que esos parámetros son (solamente) de un documento de expertos *(Congresista 2011-2016).

Además, tras la aprobación de la Ley, los parámetros técnicos fueron cambiando en la elaboración de distintos documentos elaborados por el MINSA, algunos de los cuales fueron sometidos a consulta pública (RM N° 321-2014-MINSA, DS N° 007-2015-SA, RM N°524-2016-MINSA y DS N° 017-2017-SA). Los entrevistados comentaron que estos cambios se debieron a la falta de parámetros técnicos oficiales de la OPS, a la falta de consenso político y a la falta de recursos económicos para hacer investigaciones que permitieran la elaboración de parámetros técnicos propios.

En 2015, la OMS publica el Modelo de Perfil de Nutrientes, el cual, de acuerdo con una ex funcionaria del MINSA, fue incluido en la propuesta de Reglamento del año 2017. Sin embargo, indicó que esta propuesta no lograba la aprobación de la PCM, por lo que se decidió optar por los parámetros técnicos chilenos que eran más laxos.

*La PCM no iba aprobar jamás una propuesta de Decreto Supremo [Reglamento] con los parámetros de OPS. Entonces, por eso es que se puso este proceso gradual [parámetros chilenos], o sea, o avanzamos o avanzamos* (Funcionaria del MINSA).

### Advertencias publicitarias

La Figura 2 describe los cambios en las AP presentes en los diferentes documentos revisados.

**Figura 2:** Cambios en el texto a incluir en las advertencias publicitarias o las indicaciones para su uso.

Una de las modificaciones más importantes se dio en el texto que tienen las AP. En el proyecto de Ley 1038/2011-CR tenían un texto extenso, que indicaba las consecuencias de consumir altas cantidades de nutrientes críticos, mientras en la Ley aprobada tenían un texto más reducido. 

Posteriormente, entre el proyecto del MAP y el MAP aprobado, se realizaron varias modificaciones, como cambiar el octógono de rojo a negro, retirar la frase «Ministerio de Salud» de las AP y cambiar el tamaño mínimo que debían tener los envases para colocar AP, de 20 cm² a 50 cm² de área de la cara frontal del envase. Una funcionaria del MINSA señaló que los cambios se dieron como respuesta a las recomendaciones que el MINSA recibió tras la prepublicación del proyecto del MAP, así como por negociaciones en la PCM, donde «no primaban únicamente los aspectos técnicos». Asimismo, señaló que el cambio de color del octógono se debió a que algunos países rechazaban el color rojo ya que representaba «peligro» y eso constituía un «obstáculo al comercio».

*El Manual de advertencias ha tenido aspectos técnicos, básicamente, pero también ha habido negociaciones… No, no lo llamaron negociaciones, sino decisiones finalmente de Alta Dirección y con un tinte más político, digamos, ¿no? O sea, no todo era resuelto finalmente por el equipo técnico* (Funcionaria del MINSA).

Además, aunque ni la Ley ni su Reglamento lo indicaban, el MAP dio un plazo de seis meses para colocar las AP con adhesivos. Más aun, este tiempo se amplió en los posteriores cuatro documentos modificatorios aprobados entre 2019 y 2022. El uso de adhesivos y su ampliación se debió a pedidos de la industria, que señalaba que poner los octógonos en las etiquetas era costoso, que no se podía exigir octógonos a productos importados, que otros países con AP permitían su uso indefinido, y que se hiciera por la pandemia.

*Yo estuve en una reunión con la industria, y nos decían que era súper caro poner la etiqueta [octógono] en la envoltura (Representante de la sociedad civil).*

*Poquito (después) de empezar la pandemia, la industria aprovechó y pidió al Ministerio de Salud que le diera un año más para usar stickers [adhesivos] en lugar de que el sello esté impreso en el empaque, ¿no? Y se aceptó *(Investigador independiente).

Finalmente, se identificaron continuas modificaciones y ampliaciones en la lista de productos exentos de usar AP, desde que fueron mencionadas por primera vez en el texto sustitutorio, previo a la aprobación de la Ley. La primera excepción a las AP fue a los ABNA en estado natural, no sometidos a proceso de industrialización. Posteriormente, en los documentos elaborados entre 2014 y 2022, se agregaron más excepciones: alimentos de procesamiento primario o mínimo, alimentos de preparación culinaria, ingredientes culinarios, sucedáneos de leche materna, alimentos de regímenes especiales sujetos al *Codex Alimentarius* y alimentos infantiles a base de cereales para niños mayores de 2 años que no tengan adición de azucares.

## DISCUSIÓN

Este breve artículo evidencia los cambios más importantes que ha tenido, en una década, la regulación de la publicidad de ABNA, las AP y los parámetros técnicos de nutrientes críticos en Perú. Los cambios identificados revelan el carácter dinámico del desarrollo de las políticas de salud ^15^, pero también que una constante en el desarrollo de esta política fue la oposición de la industria, que influyó en el Congreso y el Ejecutivo para retrasar su implementación y flexibilizar su regulación. 

La intervención de la industria alimentaria en el desarrollo de políticas públicas es frecuente en América Latina, donde su poder económico les permite influir en los actores encargados de la toma de decisiones para retrasar, modificar o denegar las regulaciones ^16^. La evidencia sugiere que utilizan estrategias similares en diferentes países de la región como es en el caso de Chile, Ecuador y México donde la industria negoció con congresistas y decisores del gobierno ^16^ ^,^ ^17^.

Durante el diseño de la Ley, la regulación de la publicidad de ABNA fue el tema que sufrió mayor oposición de la industria y los medios, provocando diversas modificaciones. El retiro de la prohibición del uso de dibujos animados o ficticios en la publicidad de ABNA fue una pérdida importante, porque es una estrategia habitual para interesar al público infantil en productos no saludables ^18^. En opinión de algunos, estas modificaciones debilitaron la regulación, pero permitieron tener apoyo suficiente en el Congreso para aprobar la Ley. No obstante, países como Chile y México pudieron mantener las prohibiciones sobre el uso de dibujos o personajes animados en los empaques de los productos con uno o más octógonos ^17^ ^,^ ^19^ ^,^ ^20^, una regulación que el Perú podría también implementar. 

En el caso de los parámetros técnicos, identificamos que un problema importante para su definición y posterior uso en las AP fue la disposición de la Ley de requerir parámetros de la OMS/OPS en un momento en el que no se contaba con ellos. Si bien los autores de la Ley hicieron esto para evitar que se utilizaran parámetros favorables para la industria y no para la salud de la población, la inexistencia de parámetros oficiales de la OMS/OPS al momento de aprobar la Ley, fue usada como argumento de la oposición para evitar su aplicación. Asimismo, identificamos que la falta de consenso dentro de la PCM finalizó con la aprobación de parámetros técnicos considerados por los defensores de la Ley como “más flexibles” que los propuestos por la OPS/OMS (Perfil de nutrientes) ^21^ ^,^ ^22^. Esta situación evidencia las limitaciones para asegurar la implementación de las políticas públicas cuando aún no se cuenta con los sustentos técnicos necesarios. Más aún, la aprobación del Reglamento con parámetros distintos a los de OMS/OPS, evidencia que, aún contando con la información requerida en la legislación (en este caso, los parámetros de la OMS/OPS indicados en la Ley), la aprobación de las políticas requiere el consenso entre los distintos actores políticos. 

Las advertencias publicitarias fueron otro punto de controversia en el desarrollo de la política, lo que se expresó tanto en la falta de consenso como en cambios constantes de su diseño. Uno de los cambios más importantes fue la modificación en el tamaño mínimo que debía tener la etiqueta para colocar las AP. Esto permitió que muchos productos con un área menor a 50 cm² no lleven octógonos ^23^ y que otros redujeran ligeramente el tamaño de sus empaques para evitar llevarlos, impidiendo así que los habituales consumidores de estos productos, es decir los niños y las niñas, tomaran decisiones de compra más informadas. Además, la experiencia mexicana ha demostrado que es posible indicar el número de octógonos de un producto por más pequeño que resulte su empaque.

En cuanto a los cambios en el diseño, inicialmente, varios promotores del uso de las advertencias apoyaban el uso del semáforo nutricional ^24^ ^,^ ^25^, sin embargo, tras la aparición del octógono, cambiaron su posición en favor de este último ^26^. Esto refleja que la evidencia científica puede ir variando en el tiempo, lo que hace que las políticas sean perfectibles y deban ser evaluadas ^27^.

Las modificaciones descritas evidencian el desarrollo de los tres componentes priorizados, cuyos cambios se explican no solo por razones técnicas sino también por negociaciones de carácter político y económico. Estos cambios implicaron que la implementación de estos componentes se retrasara hasta por seis años, como sucedió con la aplicación de las AP. Además, observamos que los documentos finalmente aprobados (Ley N° 30021, Reglamento y MAP) terminaron siendo más flexibles y potencialmente menos efectivos. Tal es el caso de las regulaciones a la publicidad, donde se retiran elementos que los autores de la Ley consideraban fundamentales; o en las AP, donde se eliminan características que, como la frase «Ministerio de Salud», podrían haber dado mayor fortaleza a las advertencias. No obstante, si bien algunas de las modificaciones debilitaron la política y redujeron su impacto, también hicieron posible que, en última instancia, pudiera ser aprobada e implementada. 

Asimismo, observamos que una limitación común y transversal a los distintos componentes analizados, fue la falta de investigación local para sustentar las decisiones de los actores en favor de la Ley. Por ejemplo, la mayor producción científica en el país hubiese ayudado a dar un mejor sustento sobre la importancia de su implementación y usarlo como argumento en contra de los opositores que sostenían que la norma era innecesaria. Más aún, las investigaciones y evaluaciones en curso de estas políticas, tanto en el Perú como en la región ^28^ ^,^ ^29^, deberían ser el insumo para realizar mejoras y fortalecerlas, acercándolas al logro de sus objetivos.

Son limitaciones del estudio solo analizar documentos de acceso público, ya que podría no haberse accedido a otras versiones con posibles modificaciones de interés; así como analizarlos exclusivamente desde una mirada de salud pública, ya que puede haber limitaciones en la interpretación de los textos a nivel legal. 

En conclusión, a la luz de sus documentos normativos, el proceso de diseño de la actual política de alimentación saludable del Perú se vio afectado por constantes demoras y por modificaciones regulatorias importantes, que habrían retrasado y reducido su impacto, y que estarían asociadas a la oposición e influencia de la industria alimentaria, la falta de consenso entre actores políticos, y a la falta de evidencia científica oportuna.
